# A Newly Established Fin Cell Line from Tomato Hind (*Cephalopholis sonnerati*) and Transcriptome Profiling Characterizing Its Response to *Vibrio harveyi* Infection

**DOI:** 10.3390/ani16203295

**Published:** 2026-10-20

**Authors:** Chongwei Wang, Chaowei Li, Youfeng Cai, Fei Fang, Qiaomu Hu, Lihong Zhang, Yong Zhang, Songlin Chen

**Affiliations:** 1 Guangdong Provincial Key Laboratory for Aquatic Economic Animals, Institute of Aquatic Economic Animals, School of Life Sciences, Sun Yat-Sen University, Guangzhou 510275, China; wangchw5@mail2.sysu.edu.cn (C.W.); lichw33@mail2.sysu.edu.cn (C.L.); caiyf33@mail2.sysu.edu.cn (Y.C.); zhlih@mail.sysu.edu.cn (L.Z.); 2 State Key Laboratory of Mariculture Biobreeding and Sustainable Goods, Yellow Sea Fisheries Research Institute, Chinese Academy of Fishery Sciences, Qingdao 266071, China; flyfang1102@126.com (F.F.); hqmu0806@163.com (Q.H.); 3 Hainan Fisheries Innovation Research Institute, Chinese Academy of Fishery Sciences, Sanya 572024, China

**Keywords:** *Cephalopholis sonnerati*, fin cell line, *Vibrio harveyi*, immune response

## Abstract

The tomato hind (*Cephalopholis sonnerati*) is an emerging mariculture species increasingly threatened by *Vibrio harveyi* infection, yet in vitro tools for studying its immune defense remain limited. Here, we established a novel fin-derived fibroblast-like cell line, designated Tomato hind Grouper Fin (TGF), from *C. sonnerati* using the tissue explant method. TGF cells have been stably subcultured for more than 83 passages in Leibovitz’s L-15 medium supplemented with 20% fetal bovine serum at 28 °C. Species authentication via *mt-co1* barcoding confirmed 99.9% sequence identity, and karyotype analysis revealed a diploid chromosome number of 2n = 48, matching the donor species. TGF cells exhibited robust post-thaw viability (>80%) and were amenable to exogenous nucleic acid delivery, with higher efficiency for siRNA than plasmid DNA. To evaluate immune competence, TGF cells were challenged with *V. harveyi*, and transcriptomic profiling was performed at 60, 90, and 120 min post-infection. A total of 1876 differentially expressed genes were identified at 120 min. KEGG enrichment analysis revealed sustained activation of Toll-like receptor, NOD-like receptor, NF-κB, TNF, and IL-17 signaling pathways across all time points. The TGF cell line represents a valuable in vitro resource for studying host–pathogen interactions of *C. sonnerati*.

## Simple Summary

The tomato hind (*Cephalopholis sonnerati*), a promising candidate for mariculture, faces growing challenges from bacterial pathogens, particularly *Vibrio harveyi*. In this study, a continuous fin-derived cell line was established from tomato hind. These cells have been stably cultured for more than 83 passages and exhibit typical fibroblast-like morphology. The cell line exhibits robust viability post-thaw and is transfectable. Transcriptomic profiling following *V. harveyi* challenge revealed the activation of multiple innate immune signaling pathways, indicating that this newly established cell line provides a valuable platform for studying host–pathogen interactions.

## 1. Introduction

Groupers, of which 47 species have been experimentally cultured or trialed for aquaculture, rank among the most valuable marine food fish traded in tropical and subtropical markets; however, only a handful are currently suitable for large-scale commercial farming [1]. The tomato hind (*Cephalopholis sonnerati*), a member of the *Serranidae* family (subfamily *Epinephelinae*), is a benthic coral reef fish native to the Indo-Pacific [2] and Red Sea regions [3]. In recent years, it has emerged as a promising candidate for mariculture diversification in China and Southeast Asia. The recent availability of genomic resources [4] and advances in hatchery rearing techniques [5] have further accelerated the domestication and commercial cultivation of this species. However, like other groupers, *C. sonnerati* is highly susceptible to bacterial diseases, particularly vibriosis caused by *Vibrio harveyi*, a Gram-negative bacterium that is ubiquitous in warm marine waters and is a well-documented opportunistic pathogen of cultured marine fish [6,7,8]. Despite the growing economic importance of *C. sonnerati*, the molecular mechanisms underlying its immune defense against bacterial pathogens remain poorly understood, largely due to a lack of suitable in vitro research tools.

Continuous cell lines derived from teleost tissues provide powerful in vitro platforms for studying host–pathogen interactions, enabling controlled functional assays that are challenging to conduct in vivo [9,10]. Recently, three continuous cell lines have been established from *C. sonnerati*: TGGO (tomato hind grouper gonad, ovarian fibroblast-like) [11], TGGI (tomato hind grouper intestine, intestinal epithelial-like) [12], and TGB (tomato hind brain, brain fibroblast-like) [13]. TGGO was challenged with infectious spleen and kidney necrosis virus (ISKNV), revealing enrichment of ECM–receptor interaction and PI3K–Akt signaling pathways; TGB was infected with nervous necrosis virus (NNV), which triggered necroptosis and cytokine–cytokine receptor interaction pathways; and most relevantly, TGGI was exposed to *V. harveyi*, with transcriptome profiling identifying the involvement of interleukin-17 (IL-17), nuclear factor kappa-light-chain-enhancer of activated B cells (NF-κB), and Toll-like receptor (TLR) signaling cascades. The TGGI study provided the first transcriptomic evidence of *C. sonnerati* cell-autonomous immune responses to bacterial challenge. However, all three existing cell lines were derived from internal organs, and none originated from the fin. As *V. harveyi* is a waterborne pathogen that first contacts fish through external body surfaces, a fin-derived cell line is essential for modeling the early-stage cutaneous immune response at the pathogen entry interface—a physiological context that internal-organ lines such as TGGI, despite their value in revealing systemic immune pathways, cannot replicate.

Fin tissue is a preferred source for establishing teleost cell lines—contributing 62 of the 280 finfish cell lines reported between 2011 and 2020 owing to its high regenerative capacity, ease of non-lethal sampling, and robust in vitro proliferation [14]. Fin-derived cell lines from various marine teleosts have proven to be valuable tools for immunological studies. The GPF (Golden Pompano Fin) line from *Trachinotus blochii* has been shown to respond to lipopolysaccharide (LPS) stimulation, providing direct experimental evidence of the immune competence of fin fibroblasts [15]. The TOF (*Takifugu obscurus* Fin) cell line from obscure puffer was challenged with *Aeromonas hydrophila*, revealing the activation of NLR and NF-κB signaling pathways and confirming that fin fibroblasts can mount pathogen-specific innate immune responses [16]. The EFF (*Epinephelus fuscoguttatus* Fin) line from the brown-marbled grouper, a species closely related to *C. sonnerati*, demonstrated susceptibility to crustacean covert mortality nodavirus (CMNV) and has served as a viral infection model [17]. Collectively, these studies establish that fin fibroblasts are immunocompetent and participate in both antibacterial and antiviral defenses. Despite this progress, no fin-derived cell line has been developed for *C. sonnerati*, and the transcriptomic response of grouper fin fibroblasts to bacterial challenge has never been characterized.

In the present study, we aimed to establish a novel fin-derived cell line from *C. sonnerati* (designated TGF) to provide a physiologically relevant in vitro platform for investigating teleost host–pathogen interactions. To this end, the cell line was systematically characterized through species authentication, morphological assessment, and exogenous gene expression analysis. Its suitability as an infection model was subsequently evaluated by challenging the cells with *V. harveyi*, followed by transcriptomic profiling to delineate the temporal dynamics of the host immune response. This study is expected to establish a valuable experimental framework for dissecting grouper disease mechanisms, identifying candidate resistance genes, and elucidating the molecular basis of teleost mucosal immunity.

## 2. Materials and Methods

### 2.1. Fish and Bacteria

Apparently healthy *C. sonnerati*, with an average body weight of 100 g, were obtained from Wanning Linlan Aquaculture Co., Ltd. (Wanning, China) and acclimated in a recirculating aquaculture system at 26–28 °C (salinity ~30‰, continuous aeration, natural photoperiod) for 2 days before experiments, with daily feeding of a commercial pellet diet (Yuehai; Zhanjiang, China). The pathogenic *V. harveyi* strain used in this study was preserved in our laboratory [18].

### 2.2. Primary Cell Culture and Subculture

The fish were anesthetized with tricaine methanesulfonate (MS-222, Aladdin, Shanghai, China) at a final concentration of 50 mg/L, adjusted to pH 7 with sodium bicarbonate/NaOH prior to use, and surface-disinfected by spraying 75% ethanol onto the body. Fin tissues were collected aseptically and immersed in 75% ethanol for 2 min within a laminar flow hood, followed by three washes with sterile phosphate-buffered saline (PBS; 137 mM NaCl, 2.7 mM KCl, 10 mM Na_2_HPO_4_, 1.8 mM KH_2_PO_4_, pH 7.4) to eliminate residual ethanol. The excised tissues were cut into fragments approximately 1 mm^3^ and placed into a 50 mL centrifuge tube. To eliminate residual impurities, the fragments were subjected to two cycles of washing: each cycle consisted of repeated pipetting with PBS followed by centrifugation at 1000× *g* for 2 min. After discarding the supernatant, the tissue fragments were resuspended in an appropriate volume of complete growth medium, and 500 μL to 1 mL of the suspension was evenly seeded into each T25 culture flasks (Corning, NY, USA), and the flasks were incubated in a Heracell Series CO_2_ incubator (Thermo Fisher Scientific, Waltham, MA, USA) at 28 °C under atmospheric conditions. The flasks were initially laid flat for 6 to 8 h to allow tissue attachment, then flipped for an additional 6 to 8 h of incubation. The complete growth medium used in this study consisted of Leibovitz’s L-15 basal medium (Beyotime, Shanghai, China) supplemented with 20% fetal bovine serum (FBS; Gibco, Thermo Fisher Scientific, Grand Island, NY, USA), 10 ng/mL basic fibroblast growth factor (bFGF; Procell, Wuhan, China), 50 μmol/L β-mercaptoethanol (2-ME; Solarbio, Beijing, China), and 1% penicillin–streptomycin (final concentrations 100 U/mL penicillin and 0.1 mg/mL streptomycin; Beyotime, Shanghai, China). Once the tissue explants were firmly attached, the complete growth medium was replaced entirely every 3 days (4 mL fresh complete growth medium per T25 flask), and cell growth was monitored daily under an inverted microscope (CKX53, Olympus, Tokyo, Japan).

For passaging, cells at 90% confluence were washed with PBS. Subsequently, 500 μL 0.25% trypsin EDTA solution (Solarbio, Beijing, China) was added. Upon microscopic observation that the cells had contracted, rounded up, and commenced detachment, 3.5 mL of complete medium was added to terminate digestion. The cells were subcultured at a split ratio of 1:2 for expansion.

### 2.3. Cryopreservation and Recovery of Cells

The cell cryopreservation solution comprised 50% L-15 complete medium, 40% FBS, and 10% dimethyl sulfoxide (DMSO; Biosharp, Hefei, China). TGF cells at passage 35 were harvested for cryopreservation. The old medium was discarded, and the cells were washed twice with PBS, followed by digestion with 0.25% trypsin. Once the cells contracted and rounded up, complete medium was added to terminate digestion. The cell suspension was collected in a sterile centrifuge tube and centrifuged at 1000× *g* for 2 min. The supernatant was discarded, and the cell pellet was resuspended in 2 mL of cryopreservation solution and aliquoted into sterile cryovials (Corning, NY, USA). The cryovials were placed in a commercial isopropanol-based gradient freezing container (Beyotime, Shanghai, China) at −80 °C to achieve a controlled cooling rate of approximately −1 °C/min overnight, and subsequently transferred to liquid nitrogen for long-term storage.

Recovery tests were conducted at 6 months post-cryopreservation. Frozen vials were rapidly thawed in a 37 °C water bath. The cells were centrifuged at 1000× *g* for 5 min, resuspended in 1 mL of complete growth medium, and seeded into a T25 flask containing 2 mL of additional medium. Immediate post-thaw viability was assessed by Trypan blue exclusion: cells with intact membranes remained unstained, whereas membrane-compromised cells appeared blue. Viability was calculated as the percentage of unstained cells among the total cells counted using a hemocytometer. It should be noted that this assay reflects membrane integrity at the time of thawing and does not account for subsequent attachment failure or loss of proliferative capacity. After 24 h, the medium was renewed to remove non-adherent cells, and cell attachment was assessed by estimating the percentage of the flask bottom area covered by attached cells under an inverted microscope.

### 2.4. Determination of the Optimal FBS Concentration

Passage 30 TGF cells were plated at 5 × 10^4^ cells/well in 6-well plates (Corning, NY, USA). The cells were divided into four groups based on FBS concentration (5%, 10%, 15%, and 20%), with 18 replicate wells established for each group. After cell attachment, the medium was replaced with fresh medium containing the corresponding FBS concentrations, and the cells were pre-cultured overnight to adapt to these specific conditions. The medium was renewed every 3 days thereafter. Starting from the next day, three replicate wells were randomly selected from each group daily, and cells were harvested and counted to determine the effect of FBS concentration on the cell proliferation rate.

### 2.5. Chromosomal Analysis

Comparative karyotype analysis was performed between the TGF cell line and the head kidney tissue of the *C. sonnerati*. For the cell line, passage 40 TGF cells were seeded into T75 flasks (Corning, NY, USA). When the cell confluence reached approximately 70%, the medium was replaced with fresh medium containing colchicine (Solarbio, Beijing, China) at a final concentration of 0.05 μg/mL. After a 6-h incubation, the cells were harvested into a 15 mL centrifuge tube and centrifuged at 1000× *g* for 5 min. The supernatant was discarded, and the cell pellet was resuspended in 5 mL of 0.0375 mol/L KCl solution for hypotonic treatment on ice for 30 min. For karyotype analysis, the fixative solution was prepared fresh as a mixture of methanol and glacial acetic acid at a ratio of 3:1 (*v*/*v*). Subsequently, 5 mL of fixative solution was added for a 5 min pre-fixation. The cells were centrifuged at 1000× *g* for 5 min, and the pellet was resuspended in 5 mL of fresh fixative for 30 min at room temperature. This fixation procedure was repeated three times. Finally, the pellet was resuspended in 1 mL of fixative and dropped onto pre-chilled glass slides from a height of 2 m. The slides were air-dried, stained with 1× Giemsa solution (Solarbio, Beijing, China) for 45 min, rinsed with running water, and dried at 37 °C.

For the in vivo control, the fish were anesthetized with MS-222 (50 mg/L) and received phytohemagglutinin (PHA; 10 μg/g; Solarbio, Beijing, China) and colchicine (10 μg/g) injections prior to head kidney collection. Head kidney tissues were dissected and mechanically disrupted by gentle mincing and teasing in PBS. The cell suspension was filtered through a 300-mesh sieve (approximately 50 μm) to eliminate tissue debris. Filtered cells underwent hypotonic treatment, fixation, and chromosome spreading as described for the cell line samples. Metaphase spreads from all samples were observed under an inverted microscope, and chromosome numbers were counted for karyotype analysis.

### 2.6. Mitochondrial mt-co1 Gene Amplification

To confirm the species origin of the established cell line, the mitochondrial cytochrome c oxidase subunit I (*mt-co1*) gene was employed as a DNA barcode for molecular identification. Specific primers (forward: 5′-CACAAAGACATTGGCACCCT-3′; reverse: 5′-CCTCCTGCAGGGTCAAAGAA-3′) were adopted from a previous study [12]. Genomic DNA was extracted using a TIANamp Marine Animals DNA Kit (TIANGEN Biotech, Beijing, China) from TGF cells at passage 20. The quality and concentration of the DNA were assessed using a NanoDrop spectrophotometer (Thermo Fisher Scientific, Waltham, MA, USA), and the DNA was diluted to 50 ng/μL for use as the template. The PCR amplification was performed in a 50 μL reaction volume, consisting of 25 μL of 2× Taq Master Mix (Vazyme, Nanjing, China), 2 μL of DNA template, 2 μL of each primer (10 μM), and 19 μL of ddH_2_O. Amplifications were carried out on a T100 Thermal Cycler (Bio-Rad Laboratories, Inc., Hercules, CA, USA). The thermal cycling conditions were as follows: initial denaturation at 95 °C for 5 min; followed by 30 cycles of 95 °C for 15 s, 58 °C for 30 s, and 72 °C for 30 s; with a final extension at 72 °C for 5 min and a hold at 12 °C. Purified PCR products were sequenced by Jiangsu CoWin Biotech Co., Ltd. Resulting sequences were aligned using BLASTn (https://blast.ncbi.nlm.nih.gov, accessed on 10 April 2025) against the NCBI nucleotide database (nt). The search was performed using the BLASTn default parameters.

### 2.7. Appraisal of Liposome-Mediated Delivery

For transfection assays, TGF cells from passage 25 were seeded into 12-well plates (Corning, NY, USA) at a density of 1 × 10^5^ cells per well. Following 8 h of attachment at 28 °C, cells were washed with PBS and switched to antibiotic free complete medium. Transfection was performed strictly according to the manufacturer’s instructions for the Lipofectamine™ 3000 kit (Thermo Fisher Scientific, Waltham, MA, USA). In brief, 100 pmol of cy3-siRNA and 20 ng/μL of pEGFP-N3 plasmid were separately diluted in Opti-MEM™ reduced serum medium (Solarbio, Beijing, China). An appropriate volume of Lipofectamine 3000 reagent was added, and the mixture was incubated at room temperature for 5 min to form transfection complexes. The complexes were then added dropwise to the cell culture wells, and cells were incubated in Opti-MEM^TM^ at 28 °C for 6 h to enhance transfection efficiency under reduced-serum conditions. After 6 h, the Opti-MEM^TM^ was replaced with complete growth medium containing 20% FBS, and cells were further cultured for 18 h before fluorescence observation and imaging using a Nikon Eclipse Ti2 inverted fluorescence microscope (Nikon, Tokyo, Japan) with filter sets for Cy3 (excitation/emission: 550/570 nm) and EGFP (excitation/emission: 488/507 nm).

### 2.8. Stimulation by V. harveyi

For harvesting, *V. harveyi* was initially resuscitated in Marine Broth 2216 (2216E; Hopebio, Qingdao, China) at 28 °C overnight in an orbital shaker (Thermo Fisher Scientific, Waltham, MA, USA) at 200 rpm. The pre-culture was then diluted 1:100 into fresh 2216E broth and grown at 28 °C and 200 rpm until logarithmic phase (optical density at 600 nm, OD_600_ = 1.0; measured with a NanoDrop spectrophotometer). Passage 60 TGF cells were plated at 2 × 10^5^ cells/well in 6-well plates and cultured overnight to 90% confluence. The cell supernatant was discarded, and the culture medium containing the bacterial suspension was added to each well at a multiplicity of infection (MOI) of 5. Control wells received an equal volume of PBS buffer. Cell samples were collected at 60, 90, and 120 min post-infection. Briefly, the supernatant was removed, and the cells were washed twice with PBS to eliminate residual bacteria. Subsequently, 1 mL of total RNA extraction reagent (Solarbio, Beijing, China) was added to each well to lyse the cells thoroughly. The cell lysates were collected and immediately stored at −80 °C for subsequent RNA extraction.

### 2.9. Transcriptome Analysis

Poly (A)+ RNA was isolated from total RNA using oligo (dT)-attached magnetic beads. Following fragmentation of the Poly (A)+ RNA, double-stranded cDNA was generated through first-strand and second-strand synthesis utilizing random primers. The cDNA products were subsequently purified, subjected to polyadenylation, and ligated with Illumina-specific adapters to construct sequencing libraries. These libraries were sequenced on an Illumina NovaSeq X Plus platform employing a paired-end 150 bp (PE150) strategy.

Bioinformatic analysis commenced with quality control using fastp (v0.23.2) to obtain clean reads [19]. Potential rRNA contamination was filtered by aligning reads against the rRNA database using Bowtie2 (v2.5.1) [20]. High-quality reads were subsequently aligned to the *C. sonnerati* genome (GCA_043388385.1) using HISAT2 (v2.2.1) with default splice-aware parameters [21]. Transcript assembly was executed with StringTie (v2.2.1) [22], while expression levels were estimated as FPKM (Fragments Per Kilobase of transcript per Million mapped reads) via RSEM (RNA-Seq by Expectation-Maximization). We employed DESeq2 (v1.42.0) to identify differentially expressed genes (DEGs), applying thresholds of |log_2_FC| ≥ 1 and adjusted *p* ≤ 0.05 [23]. Finally, GO and KEGG pathway enrichment analyses were performed on the DEG sets to elucidate biological functions (*p* < 0.05) and results were visualized using online toolkit (https://www.omicshare.com/tools/, accessed on 21 June 2025).

## 3. Results

### 3.1. Establishment and Long-Term Culture of Primary Cells

**Figure 1:**
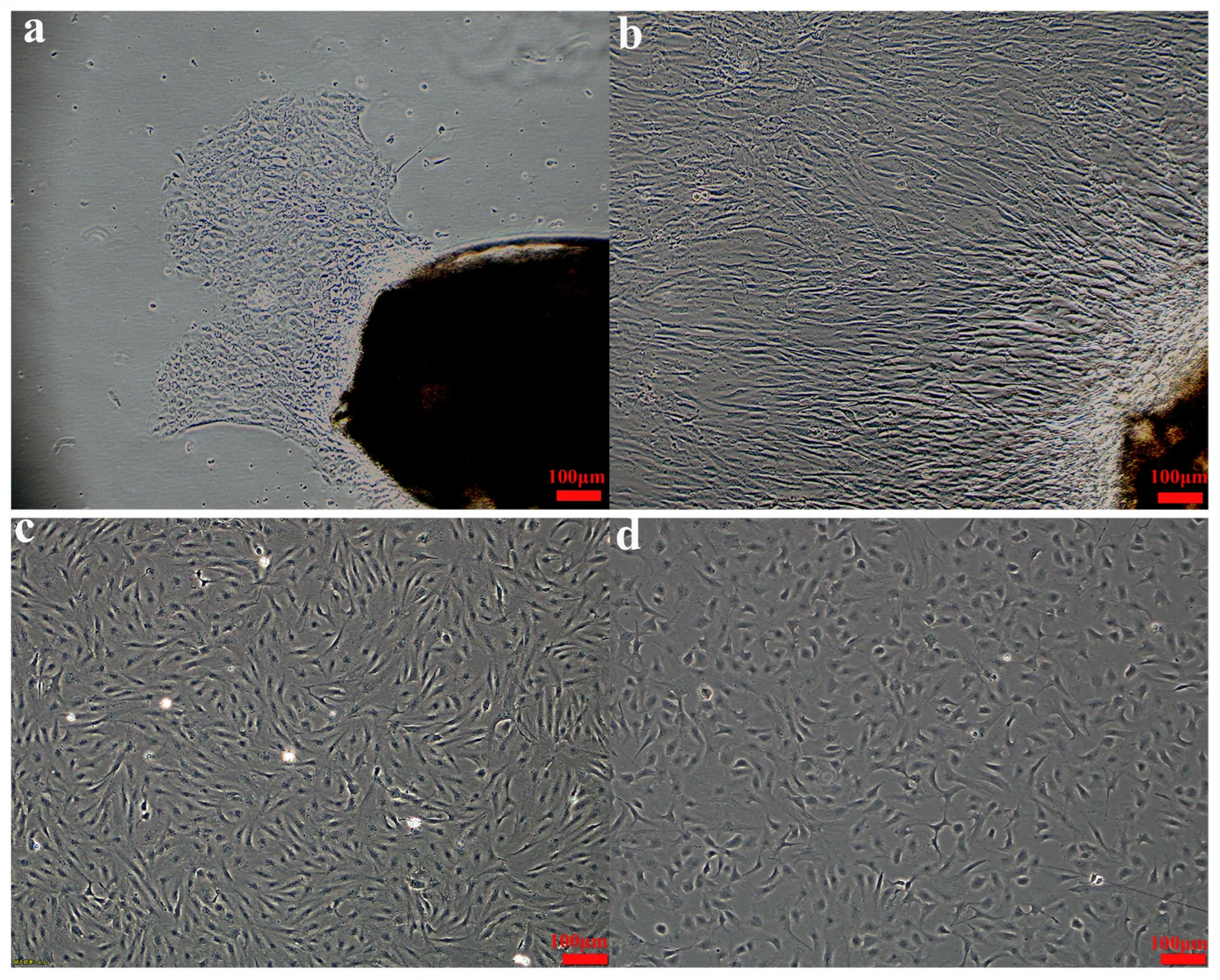
Representative micrographs illustrating the growth dynamics and morphological stability of the TGF. (**a**) Attachment and spreading of primary cells on day 4. (**b**) A continuous sheet of confluent cells resulting from the migration and spreading of primary explants. (**c**) Denser cell distribution observed at passage 64. (**d**) TGF at passage 83. Scale bars = 100 μm.

Tissue explants adhered normally to the culture surface within 24 h after seeding. Microscopic observation revealed that a small number of cells had migrated out from the edges of the fin explants by day 4 (Figure 1a). Over time, cell emigration continued to increase, and by day 22 a confluent outgrowth zone had formed. Within this zone, cells at the periphery remained as a flattened monolayer, whereas cells adjacent to the explant edges were markedly denser and exhibited multilayered growth (superimposed cell layers), as depicted in Figure 1b. Primary passage was subsequently performed. Although the larger explants remained adherent and continued to generate migrating cells after passaging, it took approximately 5–7 days for the cells to reach about 90% confluence again. With successive passaging, the cell growth characteristics gradually stabilized. By passage 20, the proliferation rate had increased significantly, and the cells displayed a typical fibroblast-like morphology, allowing for subculture every 3 days. To date, the fin-derived cell line has been stably passaged to generation 83 and is formally designated as the TGF cell line (Figure 1d).

### 3.2. Cryopreservation and Viability Assessment After Thawing

For the TGF cells cryopreserved at passage 35, recovery was assessed after 6 months of storage. Immediately after thawing, the post-thaw viability determined by trypan blue exclusion exceeded 80%. Within 24 h after seeding, approximately 60% of the flask bottom area was covered by attached cells, and these cells exhibited normal fibroblast-like morphology. The attached cells resumed proliferation at a rate comparable to that before cryopreservation and could be routinely subcultured within 3 days.

### 3.3. Determination of Optimal FBS Concentration

**Figure 2:**
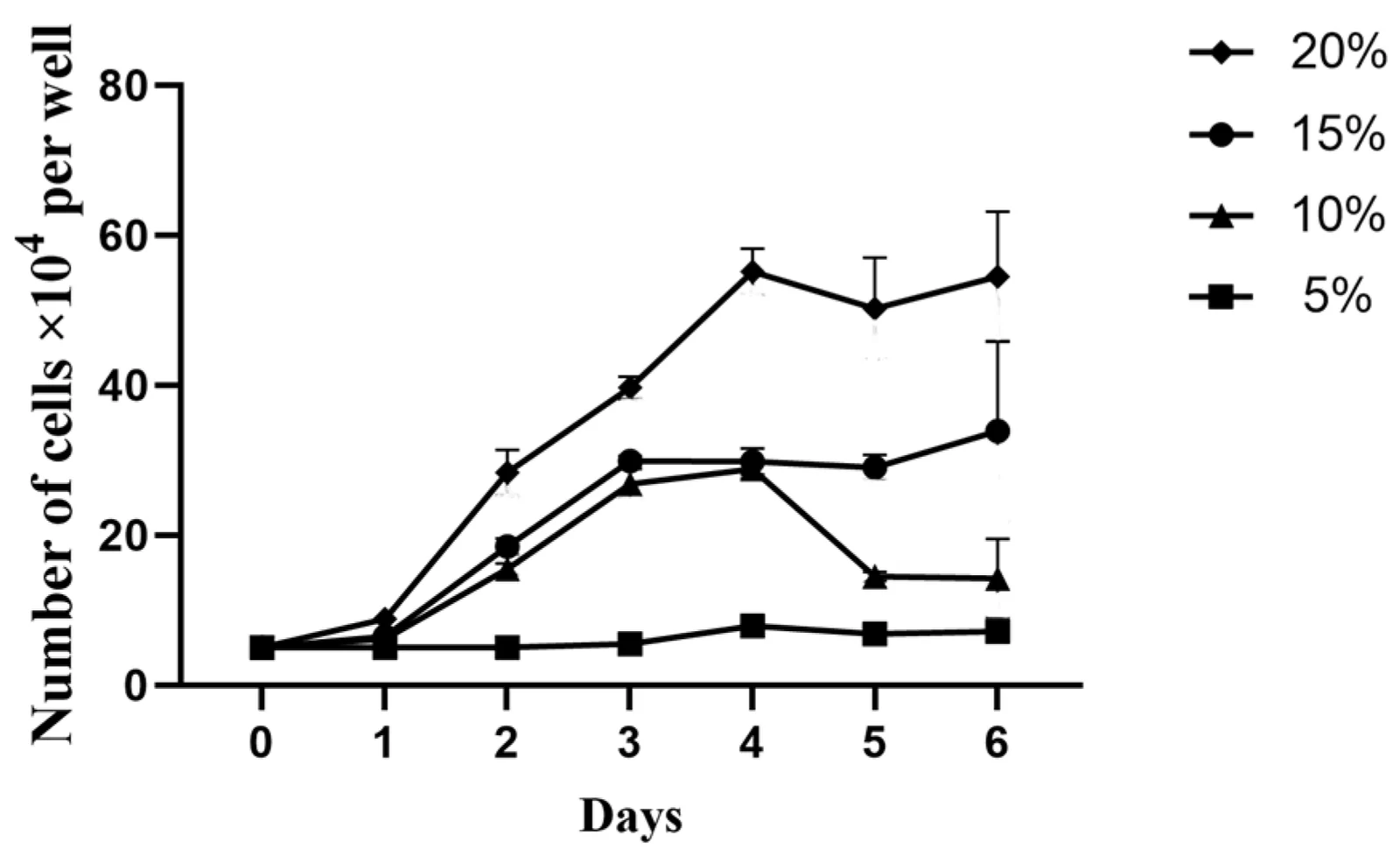
Growth kinetics of TGF cells cultured in L-15 medium supplemented with graded levels of FBS. Cell numbers are reported as total cells per well. For reference, 1 × 10^5^ cells/well ≈ 1.05 × 10^4^ cells/cm^2^ (6-well plate, 9.5 cm^2^ growth area). Data are presented as the mean ± standard deviation (SD).

Complete growth media were prepared by supplementing L-15 basal medium with fetal bovine serum at concentrations of 5%, 10%, 15%, and 20%. TGF cells at passage 30 were seeded at a uniform density of 5 × 10^4^ cells/well and cultured in these media, and the growth curves are presented in Figure 2. The curves could be broadly divided into a lag phase (Days 0–1), an exponential (log) phase (approximately Days 1–4), and a subsequent plateau or decline phase. Cell proliferation was extremely slow in the medium containing 5% FBS. To compare growth rates quantitatively, cell numbers were log_2_-transformed and the population doubling time (PDT) during the exponential phase was calculated by linear regression over Days 0–3; the PDT was 22.5 h, 25.9 h and 27.9 h for 20%, 15% and 10% FBS, respectively, whereas the apparent PDT for 5% FBS was ~113 h, indicating minimal proliferation. Thus, the proliferation rates in media with 10% and 15% FBS were comparable, whereas the fastest growth was observed in the medium containing 20% FBS. Based on these results, 20% FBS was provisionally selected for subsequent culture.

### 3.4. Karyotypic Characterization

**Figure 3:**
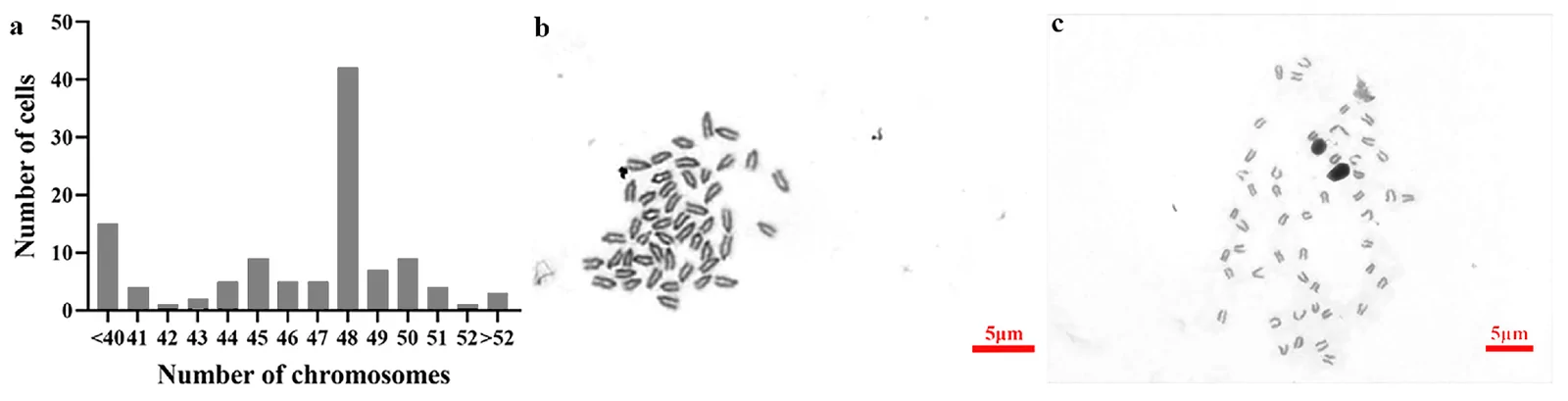
Comparative cytogenetic analysis between the TGF cell line and the native tissue of *C. sonnerati*. The scale bar represents 5 μm. (**a**) Histogram depicting the modal chromosome number of TGF at passage 40 (*n* = 112 metaphase spreads pooled from four independent experiments). (**b**) Metaphase spread and karyogram confirming the diploid profile of the TGF. (**c**) Corresponding diploid karyotype obtained from the head kidney cells of *C. sonnerati*.

Chromosomal analysis of passage 40 TGF cells revealed that the chromosome number ranged from <40 to >52 per cell in the observed fields, with the modal number being 48 (Figure 3a). A representative normal metaphase spread of the TGF cell line is shown in Figure 3b. To verify whether the chromosome number of the TGF cell line corresponds to that of the host somatic cells, head kidney tissue from the tomato hind was analyzed as a control. As presented in Figure 3c, the chromosome number of the head kidney cells was also 48, which is consistent with the result obtained from the TGF cell line.

### 3.5. Genetic Authentication and Species Origin Verification

PCR amplification of the *mt-co1* gene yielded a specific band at approximately 650 bp, as visualized by agarose gel electrophoresis (Figure S1a). Following gel extraction and sequencing, the obtained sequence was aligned with reference sequences in the NCBI database using BLASTn. The sequence similarity was 99.9%, confirming that the TGF cell line originated from *C. sonnerati* (Figure S1b).

### 3.6. Identification of Cell Type from RNA-Seq Data

**Figure 4:**
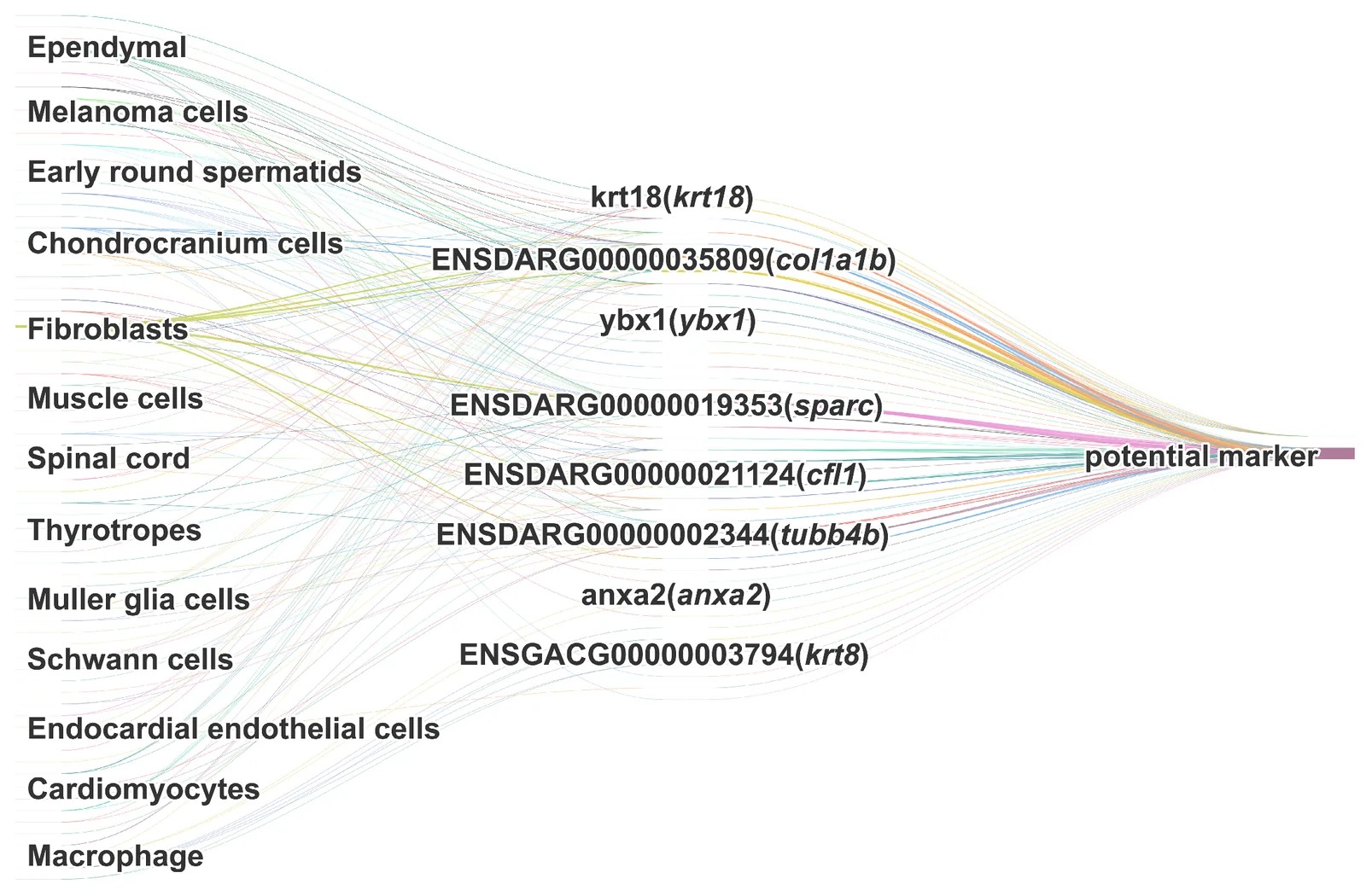
Sankey diagram showing the results of cell type identification. The top 50 highly expressed genes were queried against the Fish Single-cell RNA Database.

The top 50 most highly expressed genes were used for cell type annotation via the Fish Single-cell RNA Database (http://bioinfo.ihb.ac.cn/fishsct/multi_gene_search/, accessed on 3 October 2026; Table S1). The Sankey diagram illustrated that the cell line expresses characteristic fibroblast markers, as 15 of the 50 genes were mapped to fibroblast-associated marker genes in the database (Figure 4 and Figure S2).

### 3.7. Comparative Analysis of siRNA and Plasmid Uptake

**Figure 5:**
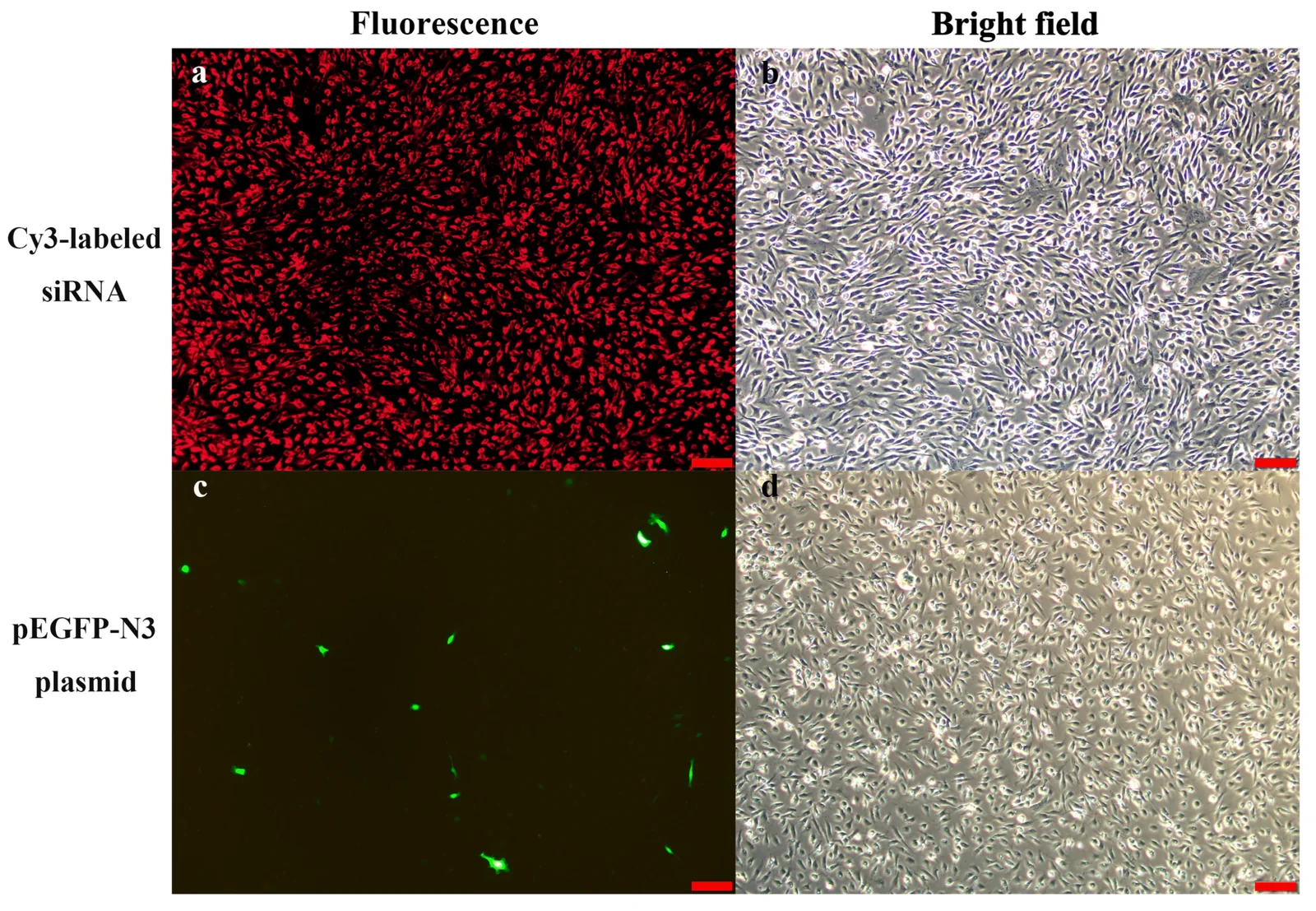
Representative fluorescence micrographs captured at 24 h following Cy3-siRNA introduction (**up**) and pEGFP-N3 delivery (**down**). (**a**) Visualization of red fluorescence emitted by Cy3-labeled scrambled siRNA at 24 h post-transfection. (**b**) Brightfield image of the same field shown in (**a**). (**c**) Expression of the EGFP reporter gene at 24 h following pEGFP-N3 plasmid delivery. (**d**) Brightfield image of the same field shown in (**c**). Scale bars = 100 μm.

Two independent delivery trials were conducted to assess the cellular response to nucleic acid introduction. When the Cy3-labeled scrambled siRNA was introduced via lipofection, robust cytoplasmic red fluorescence was evident in approximately 70–80% of cells at 24 h (Figure 5a), indicating efficient internalization. The corresponding brightfield image (Figure 5b) shows the cell morphology of the same field. Conversely, in a separate set of experiments involving the pEGFP-N3 plasmid, the expression efficiency was notably lower; only approximately 2–5% of the cell population displayed green fluorescence at the same time point (Figure 5c). The corresponding brightfield image is shown in Figure 5d. No appreciable autofluorescence was detected in untransfected control cells under identical imaging conditions.

### 3.8. Host Immune Response of TGF Cells to V. harveyi Infection

To characterize the transcriptional response of TGF cells to *V. harveyi* infection, cell samples were collected at 60, 90, and 120 min post-infection for RNA-seq analysis, with three biological replicates per group. After quality control, each library yielded 33.5–39.9 million clean reads, with Q30 values exceeding 98.9% and GC content of approximately 49% (Table S2). Clean reads were aligned to the *C. sonnerati* reference genome (GCA_043388385.1), with mapping rates ranging from 92.30% to 92.54% (Table S3).

**Figure 6:**
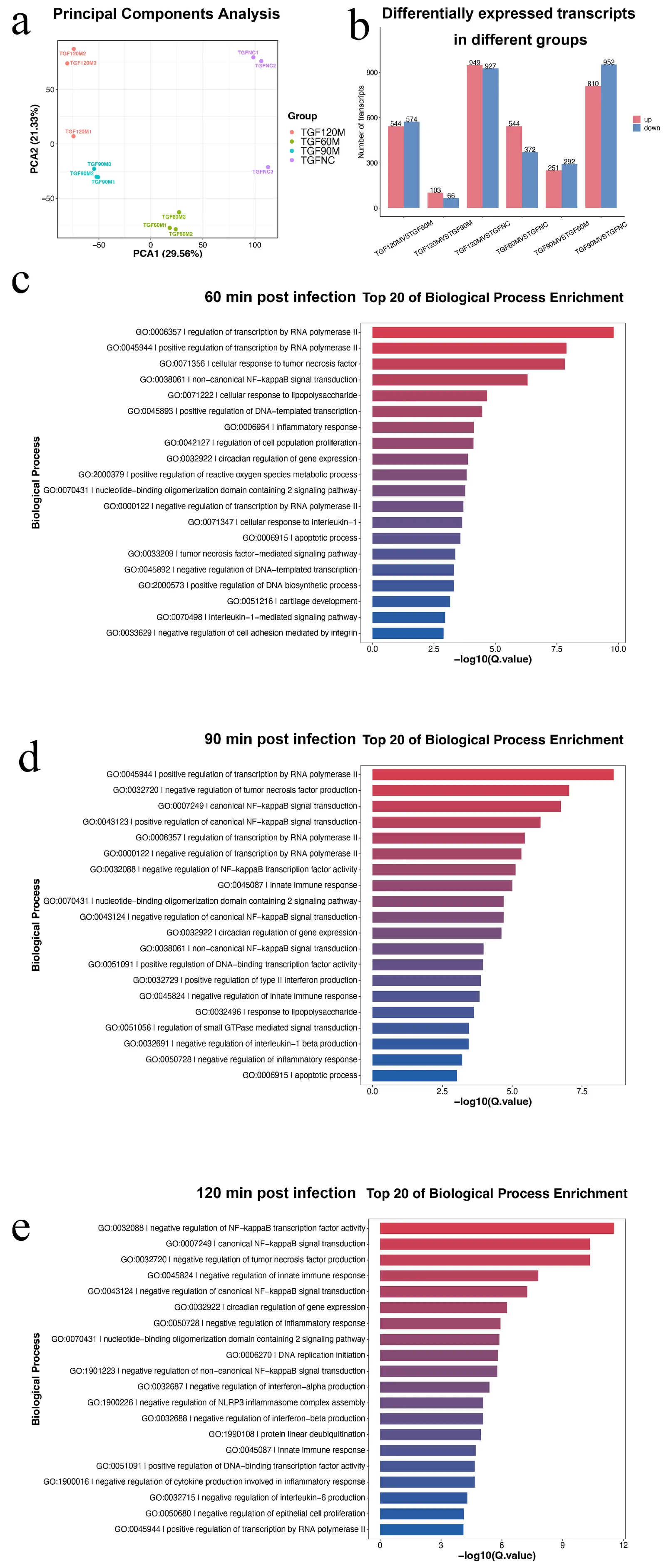
Infection challenge with *V. harveyi* and transcriptomic profiling. (**a**) Principal component analysis revealing distinct clustering of samples from different experimental groups. (**b**) Bar plot quantifying the number of up-regulated (red) and down-regulated (blue) genes across the six comparison groups. (**c**–**e**) Top 20 Biological Process (BP) enrichment terms at 60 min, 90 min post-infection and 120 min post-infection, respectively. Color represents the q-value (false discovery rate).

Principal component analysis (PCA) showed that biological replicates within each group clustered together, confirming high repeatability within each group (Figure 6a). Using thresholds of |log_2_(fold change)| ≥ 1 and adjusted *p* < 0.05, DESeq2 identified differentially expressed genes (DEGs) across six pairwise comparisons. Among these, the 120 min versus control comparison exhibited the most pronounced differences, with a total of 1876 DEGs (949 upregulated and 927 downregulated). The number of DEGs increased over time, while only 169 DEGs were detected between the 120 min and 90 min time points, indicating that transcriptional reprogramming had likely reached a near-steady state by approximately 120 min post-infection (Figure 6b). Gene Ontology (GO) enrichment analysis of DEGs in TGF cells at 60, 90, and 120 min post-*V. harveyi* infection revealed consistent enrichment of immune-related terms. At 60 min, innate immune response, inflammatory response, cytokine binding, and chemokine binding were enriched. At 90 min, positive regulation of inflammatory response, tumor necrosis factor (TNF) response, and cytokine receptor binding were prominent. At 120 min, inflammatory response, immune regulation, and immunoglobulin binding remained enriched. These results indicate sustained immune activation across the infection time course (Figure 6c–e). Overall, GO enrichment analysis revealed that the DEGs were predominantly associated with immune responses (including NF-κB signaling and interferon-mediated innate immunity), transcriptional regulation, and cellular adhesion (Figure S3).

**Figure 7:**
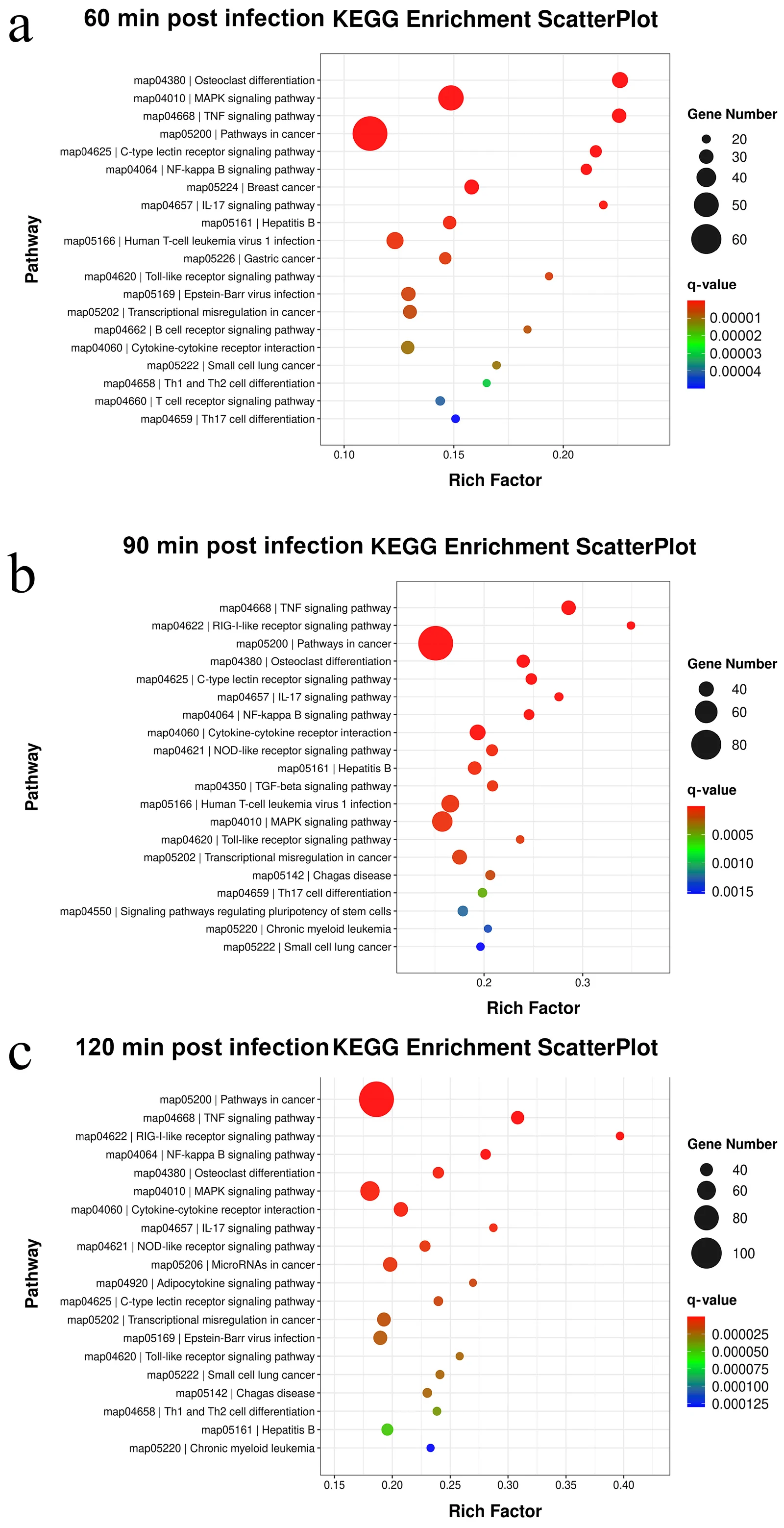
KEGG pathway enrichment analysis of DEGs at different infection time points. (**a**–**c**) Enrichment analysis at 60, 90, and 120 min post-infection, respectively. The *x*−axis represents the Rich Factor, the point size indicates the Gene Number, and the color represents the q-value (false discovery rate).

Kyoto Encyclopedia of Genes and Genomes (KEGG) pathway enrichment analysis was performed on the DEGs at three time points. At 60 min post-infection, the DEGs were predominantly enriched in pathways related to innate immune recognition and inflammation, including the TNF signaling pathway, NF-κB signaling pathway, IL-17 signaling pathway, cytokine–cytokine receptor interaction, Toll-like receptor signaling pathway, NOD-like receptor signaling pathway, and RIG-I-like receptor signaling pathway (Figure 7a). As the infection progressed to 90 min, the enrichment analysis continued to highlight the activation of core immune–inflammatory pathways. Key pathways identified included the TNF signaling pathway, NF-κB signaling pathway, cytokine–cytokine receptor interaction, IL-17 signaling pathway, Toll-like receptor signaling pathway, and NOD-like receptor signaling pathway (Figure 7b). At 120 min post-infection, the DEGs remained significantly enriched in these immune-related pathways, with the most prominent representations in the TNF signaling pathway, NF-κB signaling pathway, cytokine–cytokine receptor interaction, IL-17 signaling pathway, and Toll-like receptor signaling pathway (Figure 7c). Notably, the TNF, NF-κB, and IL-17 signaling pathways, along with cytokine–cytokine receptor interactions, were consistently enriched across all three time points, suggesting a sustained immune response (Figure S4).

## 4. Discussion

*C. sonnerati* is an emerging grouper species with considerable economic value, yet its aquaculture expansion is increasingly threatened by *V. harveyi* infection [24,25]. Cell lines derived from teleost tissues serve as powerful in vitro tools for investigating host–pathogen interactions, enabling controlled functional studies that are impractical to perform in vivo [26,27,28]. In this study, we established a novel fibroblast-like fin cell line, designated TGF, from *C. sonnerati*, and characterized its early transcriptomic response to *V. harveyi* challenge at 60, 90, and 120 min post-infection.

The TGF cell line has been stably subcultured for over 83 passages in L-15 medium supplemented with 20% FBS at 28 °C. L-15 medium is widely used for marine fish cell culture owing to its phosphate- and amino-acid-based buffering system, which maintains stable pH without requiring a CO_2_ atmosphere [29]. TGF cells exhibited optimal growth in 20% FBS, while proliferation was nearly arrested at 5% FBS. This serum dependence is comparable to other *C. sonnerati* cell lines: TGGI was cultured in 20% FBS [12], TGB in 20% FBS [13], and TGGO in 15% FBS [11], indicating that the optimal FBS concentration varies among cell lines even within the same species. The fibroblast-like morphology of TGF is consistent with fin-derived cell lines from other marine teleosts, including GPF from golden pompano (*Trachinotus blochii*) [15], TOF from obscure puffer (*Takifugu obscurus*) [16], and EFF from brown-marbled grouper (*Epinephelus fuscoguttatus*) [17]. TGF cells demonstrated robust cryopreservation tolerance with post-thaw viability exceeding 80% after six months of liquid nitrogen storage, comparable to other grouper fin cell lines such as EFF [17].

TGF cells have been continuously passaged for more than 83 generations without signs of senescence, indicating spontaneous immortalization, a common feature of fin-derived fish cell lines established by explant culture [14]. Spontaneous immortalization of fish cell lines is in some cases accompanied by aneuploidy or chromosomal instability [29,30]. Notably, TGF retained a diploid modal chromosome number of 2n = 48 that matched the donor tissue, indicating that immortalization occurred without gross karyotypic alteration—a desirable feature for a reproducible in vitro model. Although the fibroblast signature was dominant, other cell types were also detected among the top expressed genes. Because the fin is a multi-tissue organ and TGF was not subcloned, a minor fraction of other cell types (e.g., epithelial or immune cells) may remain in the culture and contribute to the observed transcriptomic response, including the enriched cytokine and chemokine signaling pathways. We therefore interpret the immune response as a property of the whole culture rather than of fibroblasts alone.

Species authentication by *mt-co1* barcoding confirmed that TGF is derived from *C. sonnerati*, consistent with the widely adopted DNA-barcoding approach for fish cell-line validation [31,32,33], and the modal diploid number of 2n = 48 matches the donor head kidney tissue and other epinepheline groupers [34,35], supporting the genomic stability of the line. Transfection experiments showed that TGF cells were permissive to Cy3-siRNA uptake with high efficiency, whereas pEGFP-N3 plasmid delivery yielded a lower transfection rate. This differential efficiency between small RNA and plasmid DNA transfection is commonly observed in fish cell lines—for instance, BTF (*Bahaba taipingensis* Fin) cells from Chinese bahaba (*Bahaba taipingensis*) [36] and GPF cells from *Trachinotus blochii* [15] similarly exhibit more efficient siRNA than plasmid delivery. The relatively low plasmid transfection efficiency represents a practical limitation for overexpression- or reporter-based gene-function studies in TGF, and may be improved by optimizing the nucleic-acid-to-lipofectamine ratio or by evaluating alternative transfection reagents. The pEGFP-N3 reporter used in this study is driven by the human cytomegalovirus (CMV) immediate-early promoter, whose activity in fish cells is highly variable and species- and cell-type-dependent [37,38]. Because this heterologous viral promoter is frequently weaker than endogenous fish promoters such as β-actin and EF-1α [37] and tends to be progressively silenced during long-term or stable expression [38], the low plasmid-derived fluorescence observed here may partly reflect suboptimal promoter activity rather than inefficient DNA uptake. A fish-derived promoter (e.g., EF-1α) may be preferable in future assays.

Transcriptomic profiling of TGF cells following *V. harveyi* infection revealed a rapid and progressive transcriptional response. DEGs increased from 60 to 120 min post-infection, with 1876 DEGs detected at 120 min relative to controls. Notably, only 169 DEGs were identified between 90 and 120 min, suggesting that the core transcriptional program approaches a steady state by approximately 120 min post-challenge. This temporal pattern—rapid initial gene activation followed by transcriptional attenuation—is consistent with in vivo transcriptomic studies of grouper–*V. harveyi* interactions, where the majority of immune gene induction occurs within the first several hours of infection [39].

KEGG pathway enrichment analysis identified a conserved set of innate immune signaling cascades activated across all three time points. TLR signaling was prominently enriched, consistent with the established role of fish TLRs in recognizing bacterial ligands such as lipopolysaccharide, flagellin, and peptidoglycan [40,41]. NOD-like receptor (NLR) signaling was also significantly enriched—a finding not prominently reported in the TGGI intestinal cell line challenged with the same pathogen [12]. NLRs function as intracellular sensors of bacterial peptidoglycan derivatives and danger signals, and their activation has been increasingly documented in teleost antibacterial immunity [42]. Additionally, RIG-I-like receptor (RLR) and C-type lectin receptor signaling pathways were enriched, with C-type lectin receptor enrichment restricted to the 60 min time point. Although RLRs are canonically associated with antiviral RNA sensing, emerging evidence suggests their involvement in antibacterial defense through cross-talk with other pattern recognition pathways [43]. The early and transient enrichment of C-type lectin receptor signaling may reflect its role in the initial recognition of bacterial surface carbohydrates [44].

The NF-κB and TNF signaling pathways were consistently enriched across all time points, functioning as central hubs of the inflammatory transcriptional program [45]. The IL-17 signaling pathway also showed sustained activation—a feature shared with the TGGI intestinal cell line following *V. harveyi* challenge [12]. In teleosts, IL-17 signaling plays a critical role in mucosal barrier defense against extracellular bacteria [46], and its activation in both TGF (fin fibroblast) and TGGI (intestinal epithelium) suggests that IL-17-mediated antibacterial defense represents a conserved strategy across anatomically distinct barrier tissues in *C. sonnerati*. GO enrichment analysis corroborated these findings, with innate immune response, inflammatory response, and cytokine binding terms consistently enriched across time points. Compared with TGGO, which responded to ISKNV infection with ECM–receptor interaction and PI3K–Akt pathway enrichment [11], and TGB, which activated necroptosis and cytokine–cytokine receptor interaction pathways upon NNV infection [13], the *V. harveyi*-triggered transcriptome of TGF is dominated by classical innate immune activation, consistent with the distinct signaling requirements of antibacterial versus antiviral responses.

Several limitations of this study warrant consideration. First, the optimal culture temperature of TGF was not systematically investigated; given that *C. sonnerati* is a tropical species, evaluating growth across a range of temperatures (e.g., 26–32 °C) could further optimize the line for downstream applications. Second, cells in the FBS-gradient assay were adapted to each serum concentration for only a single night before growth was measured; a longer pre-adaptation period would yield more precise growth kinetics.

## 5. Conclusions

To summarize, this study successfully established a continuous fin cell line from *C. sonnerati*, named TGF, and examined its properties and applicability. TGF cells exhibited typical fibroblast-like morphology, stable proliferation over 83 passages, and robust post-thaw viability. Karyotype analysis confirmed a diploid chromosome number of 2n = 48, matching the donor species. Transcriptomic profiling following *V. harveyi* challenge revealed the coordinated activation of multiple innate immune pathways, including TLR, NLR, NF-κB, TNF, and IL-17 signaling cascades, with the latter three consistently enriched across all time points. TGF is suitable for *Vibrio*-related immunological and bacteriological assays and can express exogenous proteins. This cell line represents a valuable in vitro resource for gene function studies and investigations of host–pathogen interactions in this emerging aquaculture species. TGF has been accessioned in the National Marine Aquatic Germplasm Resource Bank (YSFRI-C-2024-TGF).

## Data Availability

Transcriptome data have been deposited in the SRA database with the accession number PRJNA1458473.

## Supplementary Materials

The following supporting information can be downloaded at: https://www.mdpi.com/article/10.3390/ani16203295/s1. Figure S1: Molecular authentication of the TGF cell line via *mt-co1* marker; Figure S2: Enlarged view of the cell type identification Sankey diagram; Figure S3: Comparative Gene Ontology (GO) enrichment analysis of biological processes across different experimental groups; Figure S4: Comparative KEGG pathways enrichment analysis across different experimental groups; Table S1: Top 50 highly expressed genes in the transcriptome data; Table S2: Summary of sequencing data quality results; Table S3: Summary of sequencing data mapping statistics.

## Abbreviations

The following abbreviations are used in this manuscript:

bFGF	Basic fibroblast growth factor
*mt-co1*	Cytochrome c oxidase subunit I
DEG	Differentially expressed gene
DMSO	Dimethyl sulfoxide
FBS	Fetal bovine serum
FPKM	Fragments per kilobase of transcript per million mapped reads
GO	Gene Ontology
IL-17	Interleukin 17
ISKNV	Infectious spleen and kidney necrosis virus
KEGG	Kyoto Encyclopedia of Genes and Genomes
MOI	Multiplicity of infection
NF-κB	Nuclear factor κ-light-chain-enhancer of activated B cells
NLR	NOD-like receptor
NNV	Nervous necrosis virus
PBS	Phosphate-buffered saline
PCA	Principal component analysis
PCR	Polymerase chain reaction
RIG-I	Retinoic acid-inducible gene I
RLR	RIG-I-like receptor
RNA-seq	RNA sequencing
TGF	Tomato hind grouper fin
TLR	Toll-like receptor
TNF	Tumor necrosis factor
2-ME	β-mercaptoethanol
