# Supplementary material for: A Newly Established Fin Cell Line from Tomato Hind (Cephalopholis sonnerati) and Transcriptome Profiling Characterizing Its Response to Vibrio harveyi Infection
Source: Animals (Basel). 2026 Oct 20;16(20):3295. doi: 10.3390/ani16203295 (PMC13700717; doi:10.3390/ani16203295)
Supplement: Supplementary file 1 [file animals-16-03295-s001.zip › animals-4556673-supplementary.pdf]

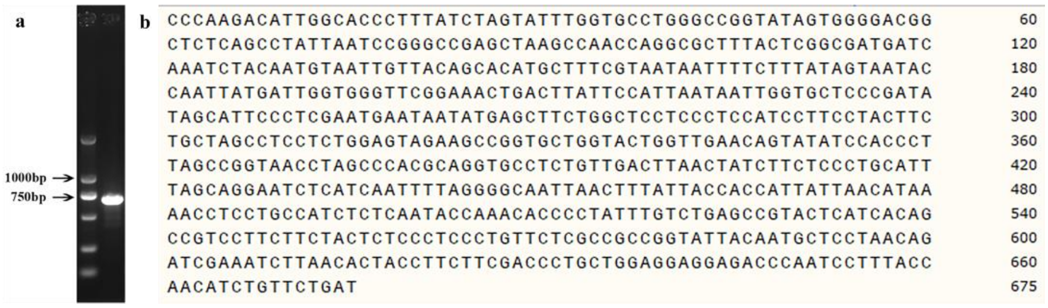


**Figure S1.** Molecular authentication of the TGF cell line via mt-co1 marker. (a) Representative image of the mt-co1 PCR product resolved by 1% agarose gel electrophoresis. Lane 1: DL2000 DNA Marker; Lane 2: mt-co1 amplicon (≈650 bp). (b) Partial nucleotide sequence of the amplified mt-co1 fragment (650 bp), aligned with the reference sequence from *C. sonnerati*.

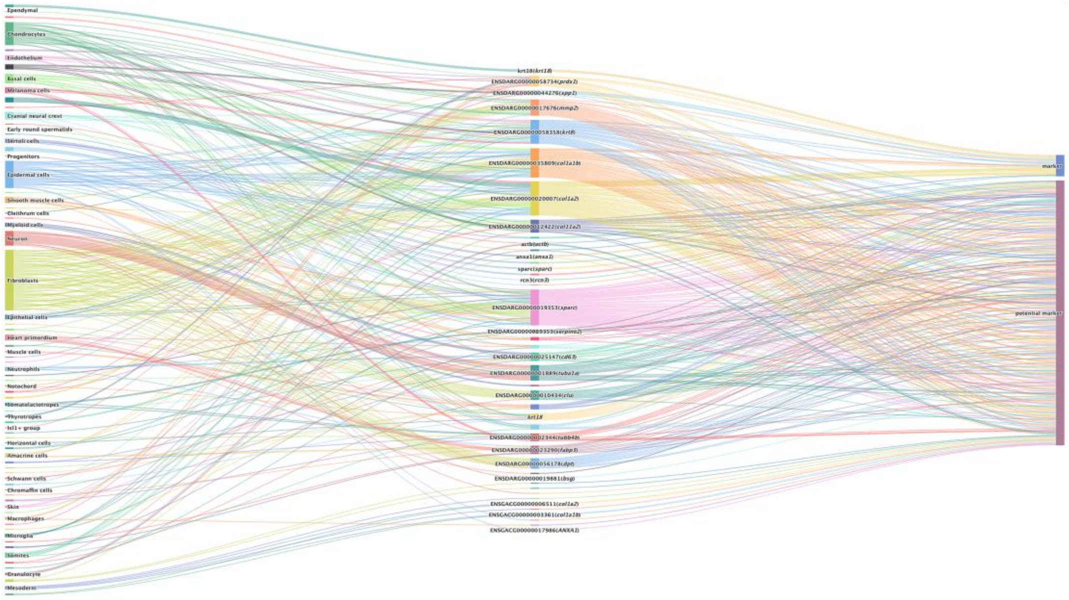

**Figure S2.** Enlarged view of the cell type identification Sankey diagram. The left column represents different cell types; the middle column represents selected genes (categorized as either markers or potential markers); and the right column represents the target markers or potential markers. The width of each flow band is proportional to the number of candidate genes.

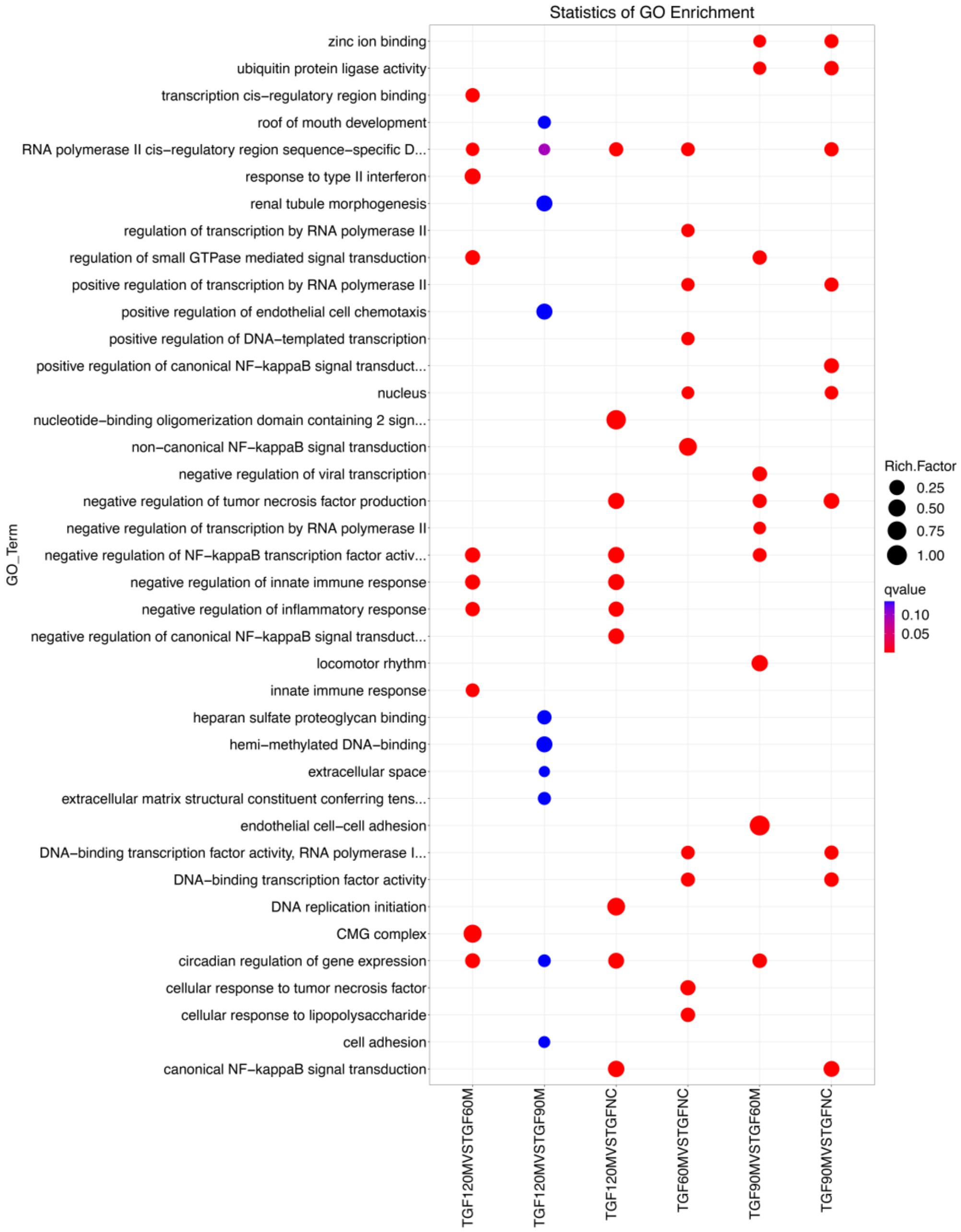


**Figure S3.** Comparative Gene Ontology (GO) enrichment analysis of biological processes across different experimental groups.

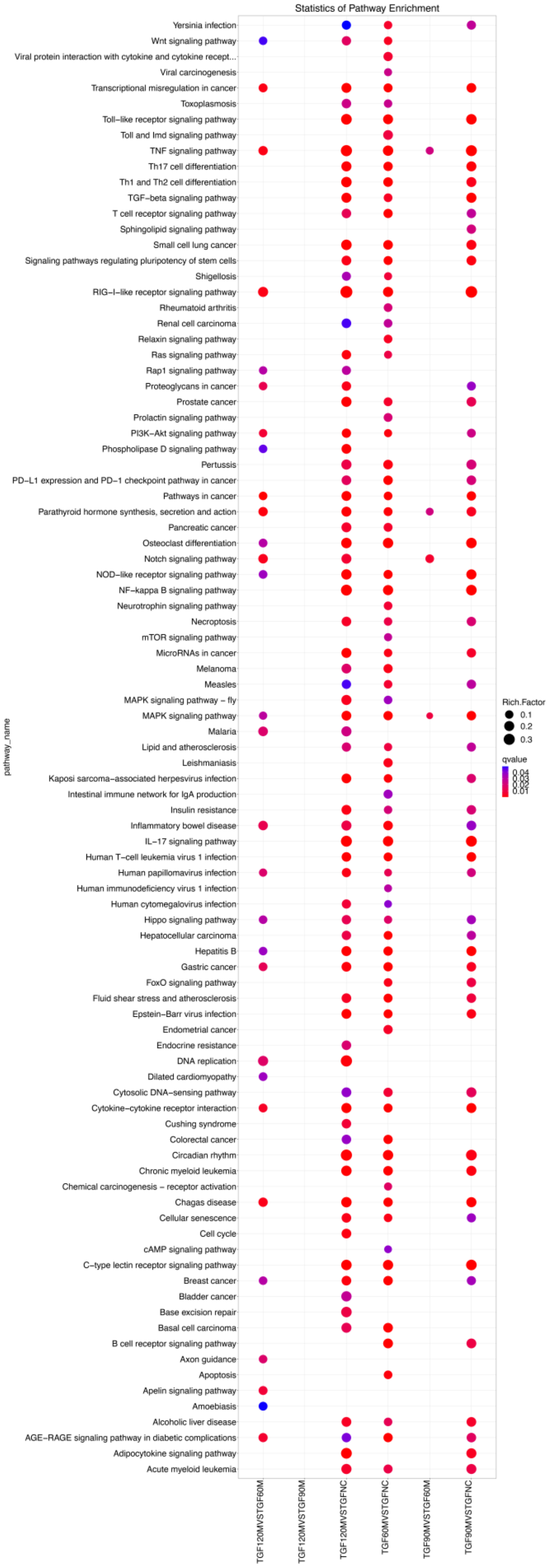


**Figure S4.** Comparative KEGG pathways enrichment analysis across different experimental groups.

**Table S1.** Top 50 highly expressed genes in the transcriptome data.

| gene_id | symbol | description | average FPKM |
|---|---|---|---|
| CsonChr16AG164890 | *actb* | *actin, cytoplasmic 2* | 5221.3 |
| CsonChr14AG145160 | *gstr1* | *rho-class glutathione S-transferase* | 5214.1 |
| CsonChr14AG144310 | *col1a2* | *collagen alpha-2(I) chain isoform X2* | 4800.7 |
| CsonChr9AG088830 | *col10a1* | *collagen alpha-1(X) chain* | 4564.3 |
| CsonChr1AG007760 | *itgb1bp2* | *muscle-specific beta 1 integrin binding protein 2 isoform x1* | 4296.7 |
| CsonChr20AG203060 | *col1a1b* | *collagen, type I, alpha 1b* | 3317.9 |
| CsonChr11AG110180 | *cr1* | *complement receptor type 1 isoform x1* | 3312.1 |
| CsonChr2AG017750 | *krt18* | *keratin, type I cytoskeletal 18-like* | 2304.1 |
| CsonChr4AG042470 | *sparc* | *secreted protein acidic and rich in cysteine* | 2304.0 |
| CsonChr19AG192960 | *spp1* | *secreted phosphoprotein 1* | 2029.9 |
| CsonChr11AG115930 | *krt8* | *keratin, type II cytoskeletal 8* | 2013.0 |
| CsonChr16AG168160 | *col1a1* | *collagen alpha-1(I) chain* | 1487.4 |
| CsonChr7AG067550 | *anxa1* | *annexin a1a* | 1352.6 |
| CsonChr13AG137300 | *serpinh1* | *serpin H1b isoform X1* | 1336.6 |
| CsonChr17AG174660 | *slc25a43* | *solute carrier family 25 member 43* | 1329.6 |
| CsonChr3AG027320 | *vav1* | *proto-oncogene vav* | 1329.0 |
| CsonChr11AG114640 | *ybx1* | *y-box binding protein 1 isoform X1* | 1300.3 |
| CsonChr9AG090960 | *clu* | *clusterin* | 1218.6 |
| CsonChr10AG098700 | *prdx1* | *peroxiredoxin-1* | 1177.0 |
| CsonChr21AG210010 | *ptgds* | *prostaglandin d2 synthase b, tandem duplicate 1* | 1157.3 |
| CsonChr18AG181110 | *p4ha1* | *prolyl 4-hydroxylase subunit alpha-1b isoform x1* | 1135.5 |
| CsonChr3AG031220 | *notum* | *carboxylesterase notum2, partial* | 951.3 |
| CsonChr14AG141190 | *spaca* | *sperm acrosome membrane-associated protein 4* | 933.3 |
| CsonChr7AG064660 | *tubb4b* | *Tubulin beta-4b chain* | 906.9 |
| CsonChr4AG042130 | *fgfbp2* | *fibroblast growth factor-binding protein 2* | 867.9 |
| CsonChr4AG037340 | *ccng1* | *cyclin-g1* | 857.0 |
| CsonChr14AG140670 | *eva1b* | *eva-1 homolog bb* | 842.0 |
| CsonChr6AG059590 | bsg | *basigin* | 828.8 |
| CsonChr14AG145310 | *s100a2* | *s100 calcium binding protein v2* | 812.1 |
| CsonChr9AG094810 | *ggct* | *glutathione-specific gamma-glutamylcyclotransferase 1* | 788.8 |
| CsonChr22AG212780 | *cd63* | *cd63 antigen-like isoform x1* | 751.1 |
| CsonChr14AG143490 | *fabp3* | *fatty acid-binding protein, heart* | 717.8 |
| CsonChr11AG113050 | *myl6* | *myosin light polypeptide 6 isoform X2* | 656.3 |
| CsonChr19AG193510 | *gsn* | *gelsolin a isoform x2* | 648.0 |
| CsonChr14AG143800 | *ndrg1* | *protein ndrg1-like protein* | 641.9 |
| CsonChr11AG112110 | *bgn* | *biglycan b* | 621.5 |
| CsonChr13AG129650 | *serpine2* | *glia-derived nexin* | 586.0 |
| CsonChr5AG047090 | *calu* | *calumenin-b* | 542.0 |
| CsonChr11AG107960 | *tuba1a* | *tubulin alpha-1a chain* | 528.2 |
| CsonChr11AG112390 | *csrp1* | *cysteine and glycine-rich protein 1a* | 518.8 |
| CsonChr16AG157830 | *rcn3* | *reticulocalbin-3 isoform X2* | 517.1 |
| CsonChr4AG040300 | *cfl1* | *cofilin-1* | 509.7 |
| CsonChr6AG055540 | *dpt* | *dermatopontin* | 504.5 |
| CsonChr24AG227950 | *tuba* | *tubulin alpha chain-like* | 497.3 |
| CsonChr1AG002250 | *mmp2* | *72 kDa type IV collagenase* | 350.3 |
| CsonChr5AG046350 | *anxa2* | *annexin A2-like* | 332.9 |
| CsonChr14AG139470 | *col11a2* | *collagen alpha-2(XI) chain isoform X3* | 325.9 |
| CsonChr17AG169740 | *p4ha2* | *prolyl 4-hydroxylase subunit alpha-2 isoform X1* | 323.2 |
| CsonChr3AG028140 | *anxa5* | *annexin a5b isoform X2* | 295.4 |
| CsonChr11AG109020 | *mmp19* | *matrix metalloproteinase-19-like* | 285.5 |

**Table S2.** Summary of sequencing data quality results.

| Sample name | Raw reads | Clean reads | GC content GC (%) | Q20 (%) | Q30 (%) |
|---|---|---|---|---|---|
| TGF120M1 | 36887970 | 35269652 | 49 | 100.00 | 98.94 |
| TGF120M2 | 36239448 | 34688602 | 49 | 100.00 | 98.96 |
| TGF120M3 | 37338852 | 35702988 | 49 | 100.00 | 98.94 |
| TGF60M1 | 41781600 | 39934690 | 49 | 100.00 | 98.97 |
| TGF60M2 | 39138004 | 37490546 | 49 | 100.00 | 98.90 |
| TGF60M3 | 34910652 | 33475088 | 49 | 100.00 | 98.96 |
| TGF90M1 | 40587558 | 38900318 | 49 | 100.00 | 99.00 |
| TGF90M2 | 35754454 | 34228978 | 49 | 100.00 | 98.94 |
| TGF90M3 | 35480734 | 34002128 | 49 | 100.00 | 98.95 |
| TGFNC1 | 41303892 | 39498268 | 49 | 100.00 | 98.97 |
| TGFNC2 | 36427040 | 34827538 | 49 | 100.00 | 98.91 |
| TGFNC3 | 36799434 | 35262734 | 49 | 100.00 | 98.91 |

**Table S3.** Summary of sequencing data mapping statistics.

| Sample | Valid reads | Mapped reads | Unique Mapped reads | Multi Mapped reads | PE Mapped reads |
|---|---|---|---|---|---|
| TGF120M1 | 35269652 | 32567906 (92.34%) | 31773054 (90.09%) | 794852 (2.25%) | 30646860 (86.89%) |
| TGF120M2 | 34688602 | 32052812 (92.40%) | 31249875 (90.09%) | 802937 (2.31%) | 30238248 (87.17%) |
| TGF120M3 | 35702988 | 33003939 (92.44%) | 32172053 (90.11%) | 831886 (2.33%) | 31137462 (87.21%) |
| TGF90M1 | 38900318 | 35937560 (92.38%) | 35031891 (90.06%) | 905669 (2.33%) | 33857746 (87.04%) |
| TGF90M2 | 34228978 | 31611952 (92.35%) | 30824628 (90.05%) | 787324 (2.30%) | 29804750 (87.07%) |
| TGF90M3 | 34002128 | 31415195 (92.39%) | 30624477 (90.07%) | 790718 (2.33%) | 29585556 (87.01%) |
| TGF60M1 | 39934690 | 36954679 (92.54%) | 35984067 (90.11%) | 970612 (2.43%) | 34920308 (87.44%) |
| TGF60M2 | 37490546 | 34668816 (92.47%) | 33792516 (90.14%) | 876300 (2.34%) | 32617372 (87.00%) |
| TGF60M3 | 33475088 | 30973108 (92.53%) | 30213388 (90.26%) | 759720 (2.27%) | 29105216 (86.95%) |
| TGFNC1 | 39498268 | 36457535 (92.30%) | 35513834 (89.91%) | 943701 (2.39%) | 34221286 (86.64%) |
| TGFNC2 | 34827538 | 32178315 (92.39%) | 31365837 (90.06%) | 812478 (2.33%) | 30288724 (86.97%) |
| TGFNC3 | 35262734 | 32612033 (92.48%) | 31775630 (90.11%) | 836403 (2.37%) | 30828778 (87.43%) |
